# The default space theory: a unified oscillatory framework for spatial orientation and balance

**DOI:** 10.3389/fnins.2026.1716799

**Published:** 2026-09-17

**Authors:** Ravinder Jerath, Varsha Malani

**Affiliations:** 1 Mind Body Technologies and Research, Augusta, GA, United States; 2 Northeastern University, Boston, MA, United States

**Keywords:** brain oscillations, consciousness, dynamic connectivity, spatial orientation, spatial representation, vestibular system

## Abstract

The Default Space Theory (DST) of consciousness holds that conscious experience arises from synchronized physiological and neural oscillations that construct an internal three-dimensional “default space” approximating the body’s position in the external world. This paper is a conjectural extension of DST to spatial orientation and postural balance. We propose that fast gamma-band oscillations (~30–100 Hz) bind visual, auditory, somatosensory, and motor input into a coherent internal spatial map, while slower thalamic theta (4–8 Hz) and alpha (8–12 Hz) rhythms provide temporal orientation—gating sensory input and permitting rapid postural correction. We synthesize anatomical, histological, and electrophysiological findings from peripheral receptors (retina, cochlea, vestibular apparatus) and central structures (cerebellum, thalamus, cortex) into a single oscillatory account, and contrast it with the conventional model, in which the cerebellum performs error-correction on vestibular and proprioceptive feedback. Under DST, thalamic integrative gating and cross-modal gamma synchrony play the larger role—a claim we argue is consistent with clinical syndromes such as vestibular migraine and sensory ataxia, where balance fails despite intact cerebellar circuitry. We outline testable predictions (e.g., increased theta-gamma coupling during active navigation) and potential interventions (e.g., 40 Hz or theta-frequency neural entrainment) that would help distinguish DST from the standard model rather than merely being compatible with it.

## 1 Introduction

Maintaining orientation and upright posture is something people do constantly without conscious effort—while standing still, walking, or dancing. That ease is itself notable: a process this automatic and this continuously error-corrected is usually assumed to require dedicated hardware, yet subjectively it requires no attention at all.

The Default Space Theory (DST), developed by Jerath and colleagues since 2015, proposes that this ease reflects a deeper property of consciousness: the brain and body continuously construct a three-dimensional internal simulation (“default space”) of the surrounding world through synchronized oscillations across sensory and motor systems (Jerath et al., 2015). In this view, the thalamus is a central integrating hub—fast oscillatory activity from the eyes, ears, and skin converges there to construct a continuously updated three-dimensional reference frame (Jerath et al., 2019a). The theory has since been extended with phenomenological analysis (Jerath et al., 2019a), a broader discussion of open questions in consciousness research (Jerath and Beveridge, 2018), and a proposed account of reflexive integration (Jerath and Beveridge, 2024).

This paper extends DST specifically to spatial orientation and balance—domains conventionally attributed to the vestibular system and cerebellum. We propose that gamma-band synchrony across visual, auditory, and somatosensory cortex constructs an internal spatial map that substitutes, moment to moment, for direct perception of the external world, and that slower thalamic rhythms (theta, ~4–8 Hz; alpha, ~8–12 Hz) provide the timing and gating needed to keep that map aligned with incoming sensory data. Hippocampal theta oscillations, already well established in encoding spatial location via place cells and grid cells (O’Keefe and Dostrovsky, 1971; Hafting et al., 2005), are proposed here to serve a parallel role in fusing visual distance information with vestibular directional cues.

This account departs from standard textbook models, which locate balance control primarily in a cerebellar reflex arc: vestibular and proprioceptive input is relayed through the brainstem to the cerebellum, which computes and corrects motor error, with the thalamus serving mainly as a relay station (Unverdi and Alsayouri, 2023). We argue this relay-only view understates the thalamus’s role. Evidence for direct thalamic integration of vestibular signals including thalamic neurons that respond to head orientation independent of cortical processing—suggests the thalamus does more than pass information along (Wijesinghe et al., 2015). Clinically, this distinction matters: patients with intact cerebellar function can still lose the ability to orient themselves after thalamic damage (Lee et al., 2005), a finding that is difficult to explain under a purely cerebellum-centered model but is predicted by a thalamus-centered one.

The chapters that follow lay out: (1) the standard model and its limitations, (2) the DST framework as applied to orientation and balance, (3) the relevant anatomy, (4) known oscillatory phenomena across each sensory system, (5) the proposed thalamic dual-oscillator mechanism, (6) cross-frequency coupling as the proposed integrating mechanism, and (7) how this account maps onto clinical vestibular and balance disorders.

## 2 Prevailing model: vestibular–cerebellar balance control

Standard accounts treat orientation and balance as a hierarchical sensorimotor reflex loop. The vestibular apparatus (semicircular canals and otolith organs) detects head movement and gravitational orientation and signals the brainstem vestibular nuclei, triggering compensatory reflexes. Proprioceptive input from muscles and joints provides parallel information about limb and trunk position. The cerebellum integrates both streams, calibrating output to correct posture and gait by modulating muscle tone and stretch-reflex gain (Unverdi and Alsayouri, 2023; Morton and Bastian, 2004). The vestibulocerebellum in particular (flocculonodular lobe) stabilizes eye movements and axial posture; damage to the cerebellar vermis produces truncal ataxia and a wide-based gait (Manto et al., 2012).

In this model, detected sway is corrected through vestibulo-spinal and stretch reflexes, with the cerebellum minimizing error iteratively. The thalamus is treated as a relay for conscious awareness of tilt or motion, not as part of the feedback loop itself (Wijesinghe et al., 2015), and multisensory (visual–vestibular) integration is generally attributed to cortical regions such as the parietal “vestibular cortex” or temporo-parietal junction.

This relay-only view is increasingly hard to square with newer findings. Vestibular nuclei project directly onto thalamic nuclei, which integrate vestibular, visual, and somatosensory input before signals reach cortex (Wijesinghe et al., 2015). In rodents, neurons in the anterior and laterodorsal thalamus encode head direction from combined vestibular and visual input—functioning as an internal compass rather than a passive relay (Wiener and Taube, 2005; Wijesinghe et al., 2015). Standing itself requires reconciling conflicting sensory streams (e.g., on a moving ship, vision reports stillness while the vestibular system reports motion), a process that appears to recruit cortical oscillatory networks alongside the cerebellum, particularly gamma-band synchrony thought to bind multisensory information (Palmisano et al., 2018).

Clinical cases sharpen the point. Thalamic stroke can produce “thalamic astasia”—an inability to maintain upright posture with no vestibular or motor deficit (Lee et al., 2005). Vestibular migraine patients show abnormal thalamic activation in response to vestibular stimulation despite normal peripheral vestibular function (Russo et al., 2014). Both are difficult to explain if the thalamus is merely a relay. We take this as motivation for a model—DST—in which the thalamus plays an active, integrative role in constructing and maintaining spatial orientation.

## 3 The default space theory framework

DST holds that consciousness and spatial self-location emerge from interconnected oscillations across brain and body, producing a “default space” that structures perception and action (Jerath et al., 2015). Gamma oscillations (~30–100 Hz) bind features within a sensory modality (e.g., color, shape, and motion in vision) and, DST proposes, also bind across modalities—for instance, aligning visual space with head orientation via visual–vestibular gamma synchrony (Jerath et al., 2019b). This produces an internally maintained three-dimensional space that persists briefly even without continuous external input (Jerath et al., 2019a)—a property DST also invokes to explain dreaming, as internally generated imagery filling the same default space in the absence of external sensory transduction.

DST assigns the thalamus an organizing role via two rhythms:

Theta (~4–8 Hz): linked to anterior thalamic and hippocampal circuits, proposed to provide a recurring “timestamp” that aligns incoming vestibular and proprioceptive signals with the current internal spatial model.
Alpha (~8–12 Hz): linked to reticular and sensory relay nuclei, proposed to gate which sensory information reaches conscious awareness, filtering out noise (e.g., minor postural sway) so that only genuine perturbations register.

Under this framework, standing on one foot involves continuous micro-corrections that occur without conscious involvement because the thalamus is constantly comparing incoming signals against the internal model and correcting deviations before they reach awareness.

DST further proposes that this oscillatory activity is not confined to canonical “processing” regions but is distributed across cellular and tissue-level electrical signaling more broadly (Jerath et al., 2015; Jerath et al., 2019b) a claim that is, by the authors’ own framing, the most speculative part of the theory and the hardest to test directly. We flag it here as a hypothesis rather than an established mechanism.

In summary, DST proposes a layered structure: gamma-band synchrony constructs moment-to-moment spatial detail; thalamic theta and alpha rhythms provide timing and gating; and the resulting default space remains stable until a genuine perturbation (loss of balance, sudden movement) forces a correction.

## 4 Anatomical and histological foundations

### 4.1 Visual system

Retinal photoreceptors transduce light into signals that travel via the optic nerve to the thalamic lateral geniculate nucleus (LGN) and then to primary visual cortex (V1), along parallel parvocellular and magnocellular channels. Notably, only about 10% of synapses onto LGN relay cells originate from the retina; the remaining ~90% come from cortical feedback and local interneurons (Sherman, 2007)—anatomical support for the idea that the LGN is not a passive relay but shapes visual signals in light of ongoing cortical context.

### 4.2 Auditory system

Hair cells in the organ of Corti transduce sound vibration into action potentials that travel via the cochlear nuclei to the medial geniculate nucleus (MGN) and then to primary auditory cortex (A1), organized tonotopically throughout. Outer hair cells also produce active mechanical oscillations (otoacoustic emissions); cortically, gamma-band activity in A1 has been linked to auditory feature binding (Singer, 1999).

### 4.3 Vestibular system

The semicircular canals (rotation) and otolith organs (linear acceleration, tilt) transduce head movement into signals carried by cranial nerve VIII to the brainstem vestibular nuclei. From there, pathways ascend to the cerebellum (flocculonodular lobe), descend via vestibulospinal tracts to spinal motor neurons, and ascend through the thalamus—primarily the ventral posterolateral (VPL) and posterior nuclei, which also process somatosensory input (Lopez and Blanke, 2011). Head-direction cells in thalamic and limbic structures fire according to head orientation relative to gravity (Taube, 2007), and thalamic projections to parieto-insular vestibular cortex (PIVC/OP2) integrate vestibular, proprioceptive, and visual signals for real-time spatial localization.

### 4.4 Somatosensory system

Proprioceptive and cutaneous signals travel via the dorsal column–medial lemniscal pathway to thalamic VPL/VPM nuclei and then to primary somatosensory cortex (S1), providing continuous feedback on limb position and load. S1 is tightly interconnected with primary motor cortex (M1), forming a loop relevant to postural correction. Sensorimotor EEG rhythms in the alpha–beta range (~10–20 Hz, the “mu” rhythm) have been linked to body-schema representation.

### 4.5 Cerebellum

Cerebellar cortex (granule cells, Purkinje cells, climbing and mossy fiber inputs) computes motor error and projects via deep cerebellar nuclei to motor output pathways and, indirectly, to the thalamus. The cerebellum is traditionally modeled as feedforward rather than oscillatory, but cortico-muscular coherence at the tremor frequency has been documented in essential tremor, implicating oscillatory sensorimotor-cortical circuits in at least some forms of postural tremor (Hellwig et al., 2001).

Taken together, these systems show extensive anatomical interconnection particularly thalamic convergence of vestibular, somatosensory, and visual input that DST interprets as infrastructure for a unified, oscillation-based spatial representation rather than several independent feedback loops.

## 5 Oscillatory activity across sensory systems

### 5.1 Visual

V1 shows stimulus-driven gamma-band activity (~40–70 Hz), classically linked to feature binding (Gray and Singer, 1989). Retinal oscillatory potentials and LGN bursting near 40 Hz have also been documented (Llinás and Paré, 1991), and Jerath et al. (2016) proposed that fast retino-geniculo-cortical oscillations in this range help construct continuous visual perception. More directly relevant to posture: EEG studies of balance perturbation show that visually-induced destabilization (e.g., sudden visual field rotation) and physically-induced destabilization both elicit theta-band (4–8 Hz) and beta-band cortical responses, with the visual condition producing stronger occipito-parietal theta activity specifically (Peterson and Ferris, 2018). This is a more direct empirical anchor for DST’s proposed link between visual/postural theta and correction, though it describes cortical response to perturbation rather than the continuous “self-motion tracking” DST proposes, and the two should not be treated as equivalent.

### 5.2 Auditory

Gamma-band synchrony supports auditory scene analysis, and cross-modal gamma coupling with visual cortex occurs for congruent audiovisual stimuli (Rohe and Noppeney, 2018). Auditory cortex also phase-locks to slower rhythms (delta/theta, ~2–7 Hz) that track the rhythm of footsteps or speech, which DST proposes could couple with vestibular rhythms during walking or dancing.

### 5.3 Vestibular

Vestibular afferents fire in proportion to head velocity/acceleration rather than at an intrinsic frequency, but rhythmic head movement (walking, intentional oscillation) can produce theta-range firing in downstream thalamic and cortical neurons, including head-direction cells (Stackman and Taube, 1998). Postural sway itself is typically very slow (~0.2 Hz at rest), rising toward 4–6 Hz under destabilizing conditions such as standing on an unstable surface with eyes closed a shift interpreted as increased corrective activity (Loram et al., 2005).

### 5.4 Cerebellar

Purkinje cell firing can show rhythmic components (~8 Hz), and cerebello-cortical-motor network activity has been characterized using MEG-based coherence imaging, which has been used to map both physiological and pathological (e.g., Parkinsonian) oscillatory motor networks involving the cerebellum (Schnitzler et al., 2006). Transient beta-band activity (15–25 Hz) has also been reported during motor learning, consistent with a role in error signaling.

### 5.5 Somatosensory/motor

Sensorimotor cortex shows well-characterized mu (~10 Hz) and beta (~20 Hz) rhythms linked to body-schema maintenance (Jurkiewicz et al., 2006). Slower body-wide rhythms cardiac (~1 Hz) and respiratory (~0.2–0.3 Hz) also modulate cortical excitability and vestibular sensitivity to some degree, though their contribution to postural control specifically remains a much smaller and less direct effect than the sensorimotor rhythms above. More broadly, non-oscillatory neuromodulatory systems also shape the variability of ongoing cortical activity: catecholamine levels have been shown to alter both the moment-to-moment variability of cortical population activity and of perception itself (Pfeffer et al., 2018), a useful reminder that oscillatory coupling is not the only mechanism regulating cortical state relevant to perception and action.

DST’s broader claim that these frequency bands are not simply present within each modality but are phase-locked with one another via thalamic and/or hippocampal timing signals (Misselhorn et al., 2019)—is the theory’s central and most novel proposal, and also the one most in need of direct empirical testing (e.g., simultaneous intracranial recording across these systems during active balance tasks).

## 6 The thalamic dual-oscillator hypothesis

DST proposes the thalamus performs two coordinated oscillatory roles:

### 6.1 Theta-based orientation timing

Anterior and lateral thalamic nuclei, working with the hippocampal formation, generate theta-band activity well documented in navigation and memory (Buzsáki, 2005). Because thalamic head-direction cells also show theta-rhythmic modulation (Tsanov et al., 2011), DST proposes this rhythm functions as a recurring “checkpoint”—roughly every 125–200 ms at 5–8 Hz at which incoming vestibular and proprioceptive signals are compared against the current internal spatial model, with corrections issued if misalignment is detected. This proposed re-calibration process is broadly consistent with behavioral evidence that returning astronauts, whose vestibular and proprioceptive inputs have been decorrelated from normal gravitational cues for months, show measurable locomotor and balance deficits that resolve gradually as sensorimotor recalibration proceeds (Mulavara et al., 2010) though that study measured functional locomotor recovery, not thalamic oscillatory activity directly, so it is consistent with a re-calibration process in general rather than evidence for this specific theta mechanism. Clinically, thalamic deep brain stimulation for tremor sometimes also improves gait and balance (Wu et al., 2008), which is at least consistent with a role for thalamic oscillatory activity in postural control, though it does not by itself establish the specific theta-timing mechanism proposed here.

### 6.2 Alpha-based gating

Thalamic relay and reticular nuclei show alpha-band activity (8–12 Hz) associated with inhibition/gating of sensory throughput (Lopes da Silva, 1991; Lorincz et al., 2009). DST proposes this rhythm suppresses irrelevant or destabilizing sensory information for example, down-weighting visual input aboard a moving vessel so that vestibular input dominates, reducing motion sickness. This is consistent with more general findings that alpha oscillations gate attentional access to sensory streams, including direct evidence that frontoparietal cortex controls which visual information is processed via modulation of anticipatory alpha desynchronization (Capotosto et al., 2009; Voytek et al., 2015; Bonnefond and Jensen, 2015), though direct evidence for this specific balance-related gating function is still limited.

Together, DST proposes the thalamus operates bidirectionally: theta activity coordinating with brainstem and spinal input to maintain reflexive alignment, and alpha activity gating what reaches cortical awareness. This framing offers a candidate explanation for several clinical patterns: thalamic astasia (loss of the theta-based alignment mechanism despite intact cerebellar and vestibular function; Lee et al., 2005), and anterior thalamic lesions producing spatial disorientation independent of postural instability, consistent with anterior thalamus’s strong connectivity to the hippocampal navigation system (Aggleton and Brown, 1999).

We note explicitly that the more detailed proposed dynamics, e.g., gamma amplitude nested within a theta “carrier” wave, itself modulated by an alpha gating envelope are modeled on well-established theta/alpha–gamma coupling as a general communication mechanism between brain regions (Bonnefond et al., 2017; Lisman and Jensen, 2013) and on evidence that theta phase modulates gamma power during spatial tasks (Bush et al., 2017). The specific claim that this same architecture governs moment-to-moment postural correction is, at present, an extrapolation rather than a directly tested finding.

## 7 Cross-frequency coupling as an integrating mechanism

Cross-frequency coupling (CFC) the coordination of oscillations at different frequencies is DST’s proposed mechanism for binding gamma-band sensory detail to theta/alpha-timed integration. CFC is well documented in navigation, attention, and sensorimotor tasks generally (Canolty and Knight, 2010).

*Theta–gamma coupling for spatial binding* is one of the best-established examples, particularly in hippocampal place-cell sequences during spatial navigation, where theta phase organizes gamma-band ensemble activity (Lisman and Jensen, 2013; O’Keefe and Recce, 1993). Human studies show comparable theta–gamma phase coupling during spatial working memory and virtual navigation tasks (Kaplan et al., 2014; Bush et al., 2017). DST extends this established mechanism to hypothesize that ongoing postural correction works the same way each theta cycle providing a timing frame, with gamma activity filling in sensory detail within that frame. This extension is plausible given the underlying mechanism’s generality, but has not, to our knowledge, been directly tested for posture specifically.

### 7.1 Alpha/beta–gamma coupling for sensorimotor regulation

Cortico-cerebellar beta rhythms (15–25 Hz) appear to support postural stability, decreasing when a corrective movement is required and rising during stable stance (Engel and Fries, 2010). Pathological over-synchronization between cortical beta and muscle activity has been observed in some tremor-related postural disorders. Alpha–gamma coupling, separately, has a well-established role in attentional gating: increased alpha and decreased gamma in a to-be-ignored sensory channel, and the reverse in an attended one (Bonnefond and Jensen, 2015). DST proposes an analogous pattern for balance visual alpha rising (suppressing unreliable visual input) while vestibular gamma is favored when vision is unreliable, though this specific application again remains a hypothesis rather than a demonstrated finding.

### 7.2 Clinical relevance

Several disorders are consistent with a CFC-disruption account, though we present this as an interpretive lens rather than established mechanism:

*Age-related imbalance* is associated with greater reliance on visual input and altered cortical network organization during balance tasks in older adults, as shown using EEG connectome analysis (Chen et al., 2021) consistent with, though not direct proof of, a shift in cross-modal coupling with age.

*Sensory ataxia* and the positive Romberg sign (loss of balance when vision is removed) are consistent with a scenario in which proprioceptive gamma input can no longer couple with visual and vestibular gamma via a shared theta reference, though direct oscillatory evidence in this specific population is limited.

*Vestibular migraine* shows abnormal thalamic activation to vestibular stimulation between episodes (Russo et al., 2014), consistent with though not proof of a thalamic gating/coupling deficit.

### 7.3 Potential interventions

Theta-frequency transcranial alternating current stimulation (tACS) over vestibular cortex has been shown to affect balance performance in healthy participants (Holle et al., 2016), and 40 Hz sensory entrainment has shown some benefit in Alzheimer’s disease models (Adaikkan and Tsai, 2020). These findings are suggestive that frequency-specific neuromodulation can influence the systems DST describes, though whether they act via the specific coupling mechanisms DST proposes (rather than some other pathway) is not yet established and should be treated as an open empirical question, not a confirmed mechanism.

## 8 Clinical correlates

This section considers how a DST-based account might interpret several vestibular and balance disorders. These are offered as candidate interpretations consistent with DST, not as established explanations in most cases, the standard model already accounts for these findings reasonably well, and DST’s contribution would be an additional layer (the oscillatory “why”), not a replacement.

### 8.1 Vestibular loss and spatial cognition

Beyond balance, chronic vestibular loss is associated with spatial memory and navigation deficits (Smith et al., 2019). DST would interpret this as a loss of theta input needed to anchor the hippocampal-thalamic spatial map to a stable gravitational reference. Vestibular rehabilitation, which trains visual-vestibular coordination, may work partly by re-establishing cross-frequency coupling between remaining systems (Herdman and Clendaniel, 2014).

### 8.2 Sensory ataxia/Romberg sign

Patients with proprioceptive deficits (e.g., dorsal column injury) can stand with eyes open but sway or fall with eyes closed, indicating reliance on conscious visual compensation for a degraded automatic (proprioceptive) system. Balance training on unstable surfaces may build alternative oscillatory coupling pathways over time (Slobounov et al., 2000).

### 8.3 Vestibular migraine

Abnormal thalamic and cortical activation patterns even between attacks (Russo et al., 2014) are consistent with a persistently lowered threshold for treating ordinary vestibular signals as destabilizing—i.e., a gating deficit, under DST’s framing.

### 8.4 Mal de debarquement syndrome

Patients feel persistent rocking/swaying at rest, often relieved by passive motion. Neuroimaging has shown increased metabolic activity in the left entorhinal cortex and amygdala in persistent MdDS, alongside decreased prefrontal and temporal metabolism notable because the entorhinal cortex is a key hub for spatial information processing en route to the hippocampus and a major driver of brain oscillations (Cha, 2015), which fits DST’s emphasis on hippocampal-thalamic spatial oscillations. Optokinetic and vestibular readaptation approaches, including standardized rotational/optokinetic protocols, have shown clinical benefit in recent treatment guidelines (Schoenmaekers et al., 2024), consistent with a resonance/re-coupling account, though this remains an active area of research rather than a settled mechanism.

### 8.5 Persistent postural-perceptual dizziness/visually-induced dizziness

Reduced functional connectivity between thalamus and putamen has been reported in these patients (Van Ombergen et al., 2017), consistent with disrupted thalamic gating and an over-reliance on visual input for orientation.

### 8.6 Cerebellar ataxia

Classic cerebellar disorders (postural tremor, titubation) are well explained by the standard model. DST’s additional claim that poor cerebello-cortical oscillatory coupling, not just cerebellar damage per se, contributes to the subjective sense of persistent unsteadiness is plausible but has limited direct evidence to date (Ushiyama et al., 2011).

### 8.7 Pusher syndrome

Following posterolateral thalamic stroke, some patients actively push themselves toward their affected (paretic) side, as though their internal “upright” reference has shifted. DST interprets this as a shift in the thalamically anchored default space, requiring visual feedback (e.g., mirrors) to retrain orientation during rehabilitation.

### 8.8 Entrainment-based therapy directions

Rhythmic sensory stimulation auditory metronomes, vibrotactile feedback, or optokinetic patterns tuned to natural postural or vestibular resonance frequencies—is a promising, already partially supported direction (Trainor et al., 2009; Iaccarino et al., 2016 for the related gamma-entrainment literature in Alzheimer’s disease). DST provides a theoretical rationale for why frequency-matched (rather than simply intense) sensory stimulation might be more effective, but this remains to be tested directly for balance rehabilitation specifically.

## 9 Conclusion and future directions

DST offers an oscillation-based alternative to the standard reflex-arc account of orientation and balance: rather than treating posture as purely a corrective response to error signals, it proposes that the brain continuously maintains an internal three-dimensional model of the body in space, with the thalamus coordinating this model via theta timing and alpha gating, and gamma-band synchrony supplying cross-modal detail. This does not require discarding the roles of the cerebellum, vestibular apparatus, or proprioceptive system it proposes that the timing and coordination between these systems carries information not captured by studying each system in isolation.

The theory generates specific, testable predictions:

1. Theta-gamma coupling between thalamus and hippocampus should increase during active spatial reorientation, testable via intracranial recording or high-density EEG.
2. Perturbing thalamic oscillations directly (e.g., 7 Hz tACS over parietal cortex) should produce a measurable, direction-specific bias in perceived head orientation or postural angle.
3. Optogenetic or targeted stimulation of hippocampal theta or cortical gamma in animal models should produce predictable biases in spatial/postural behavior, analogous to existing findings for memory and attention.

If supported, this framework suggests balance rehabilitation could benefit from an oscillatory approach rhythmic, frequency-matched multisensory training (e.g., balance exercises paired with a metronome or virtual-reality visual-vestibular training) as a complement to standard physical therapy. It may also offer a way to stratify patients (e.g., identifying a specific alpha–gamma coupling deficit in the vestibular cortex) for targeted, rather than general, neuromodulation.

We want to be direct about the theory’s current status: DST as applied here to orientation and balance is a hypothesis built by extension from an existing, more general theory of consciousness, supported by anatomical plausibility and consistency with several clinical syndromes, but not yet directly tested against its most specific and novel claims (particularly the thalamic dual-oscillator and cross-modal CFC mechanisms). The next necessary step is empirical: simultaneous multi-region recording during active balance tasks, and targeted neuromodulation studies designed to falsify, not just illustrate, the model.

## Data Availability

The original contributions presented in the study are included in the article/supplementary material, further inquiries can be directed to the corresponding author.
